# Effect of ultrasound-guided lung recruitment to reduce pulmonary atelectasis after non-cardiac surgery under general anesthesia: a systematic review and meta-analysis of randomized controlled trials

**DOI:** 10.1186/s13741-024-00379-7

**Published:** 2024-03-27

**Authors:** Bucheng Liao, Wuhao Liao, Shuang Yin, Shujuan Liu, Xinhai Wu

**Affiliations:** 1 Department of Anesthesiology, Peking University Shenzhen Hospital, No. 1120, Lianhua Street, Shenzhen, 518000 Guangdong China; 2 Department of Anesthesiology, Shenzhen Hospital, Southern Medical University, No. 1333, Xinhu Street, Shenzhen, 518000 Guangdong China

**Keywords:** Lung recruitment maneuver, Ultrasound-guided, Atelectasis, Meta-analysis

## Abstract

**Background:**

At present, the application of bedside lung ultrasound is increasing gradually, but there is no relevant expert consensus or guidance for its evaluation in the field of perioperative anesthesia. Through this meta-analysis, we tried to determine the impact of ultrasound-guided lung recruitment maneuvers (LRM) on perioperative patients.

**Methods:**

We searched PubMed, Cochrane Library database, Embase, and Clinical Trials gov for the randomized controlled trials (RCTs) published up to December 31, 2022. The primary outcome was the incidence of postoperative atelectasis. Secondary outcomes included lung ultrasound score (LUS) and LUS of each part. A total of 443 patients were examined in nine randomized controlled trials.

**Results:**

The incidence of atelectasis after surgery in patients with ultrasound-guided LRM was less (RR 0.31; 95% CI 0.25–0.40; *p* < 0.05). The LUS (WMD − 6.24; 95% CI − 6.90–5.59; *p* < 0.05) and the LUS of each part (LUS in front lung region (WMD − 2.00; 95% CI − 2.49 to − 1.51; *p* < 0.05); LUS in lateral lung region (WMD − 2.50; 95% CI − 3.20 to − 1.80; *p* < 0.05); LUS in posterior lung region (WMD − 3.24; 95% CI − 4.23 to − 2.24; *p* < 0.05)) in patients with ultrasound-guided LRM were lower.

**Conclusion:**

Ultrasound-guided lung recruitment maneuvers have been shown to be a promising approach for improving perioperative lung ventilation by increasing aeration while mitigating the development of atelectasis. In comparison to non-ultrasound-guided methods, this technique has exhibited superior effects.

**Supplementary Information:**

The online version contains supplementary material available at 10.1186/s13741-024-00379-7.

## Introduction

Atelectasis is one of the most common postoperative pulmonary complications (PPCs) of general anesthesia that occurs in patients of all ages and during all types of surgery (Forgiarini and Esquinas 2018; Fernandez-Bustamante et al. 2017). Atelectasis is a significant factor in the development of most PPCs, leading to a rise in postoperative incidence rates and mortality rates and the use of medical resources (Futier et al. 2014; Canet et al. 2010; Fernandez-Bustamante et al. 2014). With the increasing use of laparoscopic surgery, the development of pneumoperitoneum during laparoscopy and the change in body position can harm respiratory function during the procedure, which primarily worsens atelectasis during the perioperative period (Acosta et al. 2018). Therefore, preventing atelectasis during the perioperative period is a significant challenge for anesthesiologists.

Current studies demonstrate that the lung recruitment maneuver (LRM) can successfully reduce postoperative atelectasis in both children and adults (Acosta et al. 2018; Cinnella et al. 2013). The mechanism of LRM is to re-open collapsed lung units and increase end-expiratory lung volume by dynamically and instantaneously increasing trans-pulmonary pressure (Lapinsky and Mehta 2005). There are various methods of LRM, including sustained inflation, stepwise LRM through incremental PEEP, and postural LRM, among others, but the most efficient method and recruitment pressure are still unclear (Nguyen 2018; Sahetya and Brower 2017). The key reason is that LRM is not monitored in real-time, online, and intuitively. Additionally, the pressure carried by the lung unit will increase due to the increased airway pressure caused by lung re-expansion, at which point the lung unit may suffer damage from excessive expansion (Gattinoni et al. 2020). Therefore, without any image monitoring, the value of LRM is significantly reduced. Some clinical studies failed to show the benefit of the results and even produced adverse side effects (Cavalcanti et al. 2017). The benefits of LRM need to be balanced against the excessive expansion of lung units. Lung ultrasound is an easy-to-use, portable, non-invasive, visible, and radiation-free technology widely used in clinical monitoring and diagnosis (Radzina and Biederer 2019). The application in the field of perioperative anesthesia can help monitor ventilation and pathological changes. Currently, research on ultrasound-guided LRM is focused on infants and healthy adult patients (Park et al. 2021; Lee et al. 2020), confirming its feasibility. However, at present, the effect of ultrasound-guided LRM is still unclear. Differences in the incidence of postoperative atelectasis between different studies exist, and there is no consensus on whether it is worth promoting.

The primary objective of our study is to conduct a comprehensive analysis of the impact of ultrasound-guided LRM performed during general anesthesia on the development of postoperative atelectasis and lung ventilation during the perioperative period. To achieve this, we will compare its efficacy with a non-ultrasound-guided ventilation approach. The lung ventilation will be assessed using the lung ultrasound score (LUS). In order to achieve our goals, we will perform a thorough review of relevant literature, followed by a meta-analysis to establish the association between ultrasound-guided LRM and patient outcomes, such as postoperative atelectasis and LUS. Additionally, we will explore heterogeneity across studies through subgroup analyses, wherever applicable. Our endeavor is to present a scholarly and professional evaluation of the efficacy of ultrasound-guided LRM in improving patient outcomes during anesthesia.

## Materials and methods

### Agreement and registration

We present the results of this meta-analysis conducted in accordance with the preferred reporting items for systematic reviews and meta-analyses (PRISMA) guidelines (Moher et al. 2009). This study is registered in the International Prospective Register of Systematic Reviews (PROSPERO) with the registration number: CRD42023390320 (Additional file 1).

### Search strategy

The two authors (LBC and LWH) independently searched the eligible research in PubMed, Cochrane Library database, Embase, and Clinical Trials gov up to December 31, 2022. We use the medical subject (MeSH) terms to search “pulmonary atelectasis” and “ultrasound”, respectively, and add the search terms “ultrasound-guided”, ‘lung ultrasound”, and “lung ultrasound” to make the search more comprehensive. In the absence of MeSH terms related to lung recruitment, according to previous literature, we used “lung recruitment manager”, “recruitment manager”, “recruitment manager” or “RM”, “recruitment manager”, “recruitment manager”, “RM”, “open lung”, “protected exploitation” or “protective exploitation” to search (Pensier et al. 2019; Cui et al. 2020). Only randomized controlled trials were included in the study, with no language restrictions. Combining the findings from our review, we arrived at our conclusions. Table 1 displays the details of our search approach.

**Table 1:** Search strategies

String	Search
#1	ultrasonography[MeSH Terms]
#2	((((((((((((((((((((((((Diagnostic Ultrasound[Title/Abstract]) OR (Diagnostic Ultrasounds[Title/Abstract])) OR (Ultrasound, Diagnostic[Title/Abstract])) OR (Ultrasounds, Diagnostic[Title/Abstract])) OR (Ultrasound Imaging[Title/Abstract])) OR (Imaging, Ultrasound[Title/Abstract])) OR (Imagings, Ultrasound[Title/Abstract])) OR (Echotomography[Title/Abstract])) OR (Ultrasonic Imaging[Title/Abstract])) OR (Imaging, Ultrasonic[Title/Abstract])) OR (Sonography, Medical[Title/Abstract])) OR (Medical Sonography[Title/Abstract])) OR (Ultrasonographic Imaging[Title/Abstract])) OR (Imaging, Ultrasonographic[Title/Abstract])) OR (Imagings, Ultrasonographic[Title/Abstract])) OR (Ultrasonographic Imagings[Title/Abstract])) OR (Echography[Title/Abstract])) OR (Diagnosis, Ultrasonic[Title/Abstract])) OR (Diagnoses, Ultrasonic[Title/Abstract])) OR (Ultrasonic Diagnoses[Title/Abstract])) OR (Ultrasonic Diagnosis[Title/Abstract])) OR (Echotomography, Computer[Title/Abstract])) OR (Computer Echotomography[Title/Abstract])) OR (Tomography, Ultrasonic[Title/Abstract])) OR (Ultrasonic Tomography[Title/Abstract])
#3	((ultrasound-guided[Title/Abstract]) OR (lung ultrasound[Title/Abstract])) OR (lung ultrasonography[Title/Abstract])
#4	#1 OR #2 OR #3
#5	(((((recruitment maneuver[Title/Abstract]) OR (recruitment maneuvers[Title/Abstract])) OR (RM[Title/Abstract])) OR (open lung[Title/Abstract])) OR (protected ventilation[Title/Abstract])) OR (protective ventilation[Title/Abstract])
#6	pulmonary atelectasis[MeSH Terms]
#7	(((((((((((((((((((((((((((((((((((((((((((((((Atelectases, Pulmonary[Title/Abstract]) OR (Atelectasis, Pulmonary[Title/Abstract])) OR (Pulmonary Atelectases[Title/Abstract])) OR (Lung Collapse[Title/Abstract])) OR (Collapse, Lung[Title/Abstract])) OR (Atelectasis[Title/Abstract])) OR (Atelectases[Title/Abstract])) OR (Congestive Pulmonary Atelectasis[Title/Abstract])) OR (Atelectases, Congestive Pulmonary[Title/Abstract])) OR (Atelectasis, Congestive Pulmonary[Title/Abstract])) OR (Congestive Pulmonary Atelectases[Title/Abstract])) OR (Pulmonary Atelectases, Congestive[Title/Abstract])) OR (Pulmonary Atelectasis, Congestive[Title/Abstract])) OR (Congestive Atelectasis[Title/Abstract])) OR (Atelectases, Congestive[Title/Abstract])) OR (Congestive Atelectases[Title/Abstract])) OR (Atelectasis, Congestive[Title/Abstract])) OR (Resorption Pulmonary Atelectasis[Title/Abstract])) OR (Atelectases, Resorption Pulmonary[Title/Abstract])) OR (Atelectasis, Resorption Pulmonary[Title/Abstract])) OR (Pulmonary Atelectases, Resorption[Title/Abstract])) OR (Pulmonary Atelectasis, Resorption[Title/Abstract])) OR (Resorption Pulmonary Atelectases[Title/Abstract])) OR (Resorption Atelectasis[Title/Abstract])) OR (Atelectases, Resorption[Title/Abstract])) OR (Atelectasis, Resorption[Title/Abstract])) OR (Resorption Atelectases[Title/Abstract])) OR (Contraction Pulmonary Atelectasis[Title/Abstract])) OR (Atelectases, Contraction Pulmonary[Title/Abstract])) OR (Atelectasis, Contraction Pulmonary[Title/Abstract])) OR (Contraction Pulmonary Atelectases[Title/Abstract])) OR (Pulmonary Atelectases, Contraction[Title/Abstract])) OR (Pulmonary Atelectasis, Contraction[Title/Abstract])) OR (Postoperative Pulmonary Atelectasis[Title/Abstract])) OR (Atelectases, Postoperative Pulmonary[Title/Abstract])) OR (Atelectasis, Postoperative Pulmonary[Title/Abstract])) OR (Postoperative Pulmonary Atelectases[Title/Abstract])) OR (Pulmonary Atelectasis, Postoperative[Title/Abstract])) OR (Compression Pulmonary Atelectasis[Title/Abstract])) OR (Atelectases, Compression Pulmonary[Title/Abstract])) OR (Atelectasis, Compression Pulmonary[Title/Abstract])) OR (Compression Pulmonary Atelectases[Title/Abstract])) OR (Pulmonary Atelectases, Compression[Title/Abstract])) OR (Pulmonary Atelectasis, Compression[Title/Abstract])) OR (Compression Atelectasis[Title/Abstract])) OR (Atelectases, Compression[Title/Abstract])) OR (Atelectasis, Compression[Title/Abstract])) OR (Compression Atelectases[Title/Abstract])
#8	#6 OR #7
#9	((randomized controlled trial[Publication Type] OR randomized[Title/Abstract] OR placebo[Title/Abstract]) OR (RCT[Title/Abstract]))
#10	#4 AND #5 AND #8 AND #9

*Mesh* Medical Subject Headings; *[Title/Abstract]* search field; *[Publication Type]* search field; *OR* Boolean logic operator; *AND* Boolean logic operator

### Selection criteria

Two authors (LBC and LWH) independently assessed the eligibility of studies by reading the titles, abstracts, and full texts. Disagreements were resolved by the chief investigator (WXH) who made the final decisions. The following inclusion criteria were applied:

1. Design: the study had to be a human trial and only randomized controlled trials (RCTs) were considered for inclusion.
2. Age and surgery: the patients included all age groups and underwent non-cardiac surgery.
3. Intervention measures: the test group had to undergo lung recruitment strategy after ultrasound evaluation or under ultrasound guidance, while the control group either did not receive lung recruitment strategy or underwent the procedure without ultrasound guidance.
4. Eligible studies had reported postoperative atelectasis and at least one of the following outcomes: LUS and LUS of each part. These inclusion criteria were designed to ensure the validity and reliability of the study selection process.

### Results

The primary outcome was the incidence of postoperative atelectasis, while secondary outcomes included LUS and LUS of each part.

A subgroup analysis was conducted based on whether the control group used LRM, the application of positive end-expiratory pressure (PEEP) after LRM, and whether the test subjects were adults or children.

### Data extraction

The two authors (LBC and LWH) extracted the following data from the original full text: the first author, year of publication, study design, surgical type, patient characteristics [age, sex, body mass index (BMI), surgical type, sample size, ASA classification, and whether the control group uses LRM, ventilation settings (fraction of inspired oxygen (FiO_2_), tidal volume (TV), PEEP and LRM), postoperative atelectasis and LUS. Due to the difference in assessment caused by different examinations, the incidence of postoperative atelectasis here is only determined by lung ultrasound. Each hemithorax was divided into six quadrants. The intercostal space of each of the sections was scanned. Each of the 12 quadrants was assigned a score of 0 to 3 based on the following scoring system: 0, 0 to 2 B lines; 1, at least three B lines or one or more small subpleural consolidations separated by a normal pleural line; 2, multiple coalescent B lines or multiple small subpleural consolidations separated by a thickened or irregular pleural line; 3, consolidation or subpleural consolidation of more than 1 cm × 2 cm. The LUS (0 to 36) was calculated by adding the scores for the 12 quadrants, with higher scores indicating more severe aeration loss (Park et al. 2021). Any dispute shall be reviewed and decided by LSJ and WXH.

Continuous data were presented in means ± standard deviations. According to the suggestion of Cochrane Collaboration, the continuous data expressed in terms of median, interquartile range, and range are converted into mean and standard deviation (Higgins et al. 2019). If the data is only provided in graphic format, GetData Graph Digitizer 2.25 (http://getdata-graph-digitizer.com/) is adopted to quantify it.

### Statistical analysis

We followed the PRISMA standards and utilized Review Manager 5.3 (Cochrane Collaboration, Oxford, UK) and Stata 17.0 (StataCorp, College Station, TX, USA) to summarize the data according to PRISMA standards. The coefficient I^2^ is calculated to evaluate heterogeneity, which is defined as low (25–49%), medium (50–74%), and high (> 75%) levels (Higgins et al. 2003). In cases of significant heterogeneity, we conducted a meta-analysis after eliminating one study to identify the potential source. *P* < 0.05 was considered statistically significant. Egger's test was applied to evaluate the publication bias.

### Quality assessment

We employed the Cochrane Collaboration technique and evaluated methodological quality, including random sequence generation, random assignment concealment, blinding of researchers and subjects, blinding of outcome evaluators, completeness of outcome data, selective reporting of research results, and other biases. Each project contains three levels of bias risk: low bias risk, unclear bias risk, and high bias risk. We utilized GRADEpro (Schünemann et al. 2008) (McMaster University, Hamilton, Ontario, 2014), a reliable method for assessing the quality of evidence and providing recommendations for different levels of evidence. We aimed to present our results in a professional, credible, and academic manner.

## Results

### Study characteristics

**Fig. 1:**
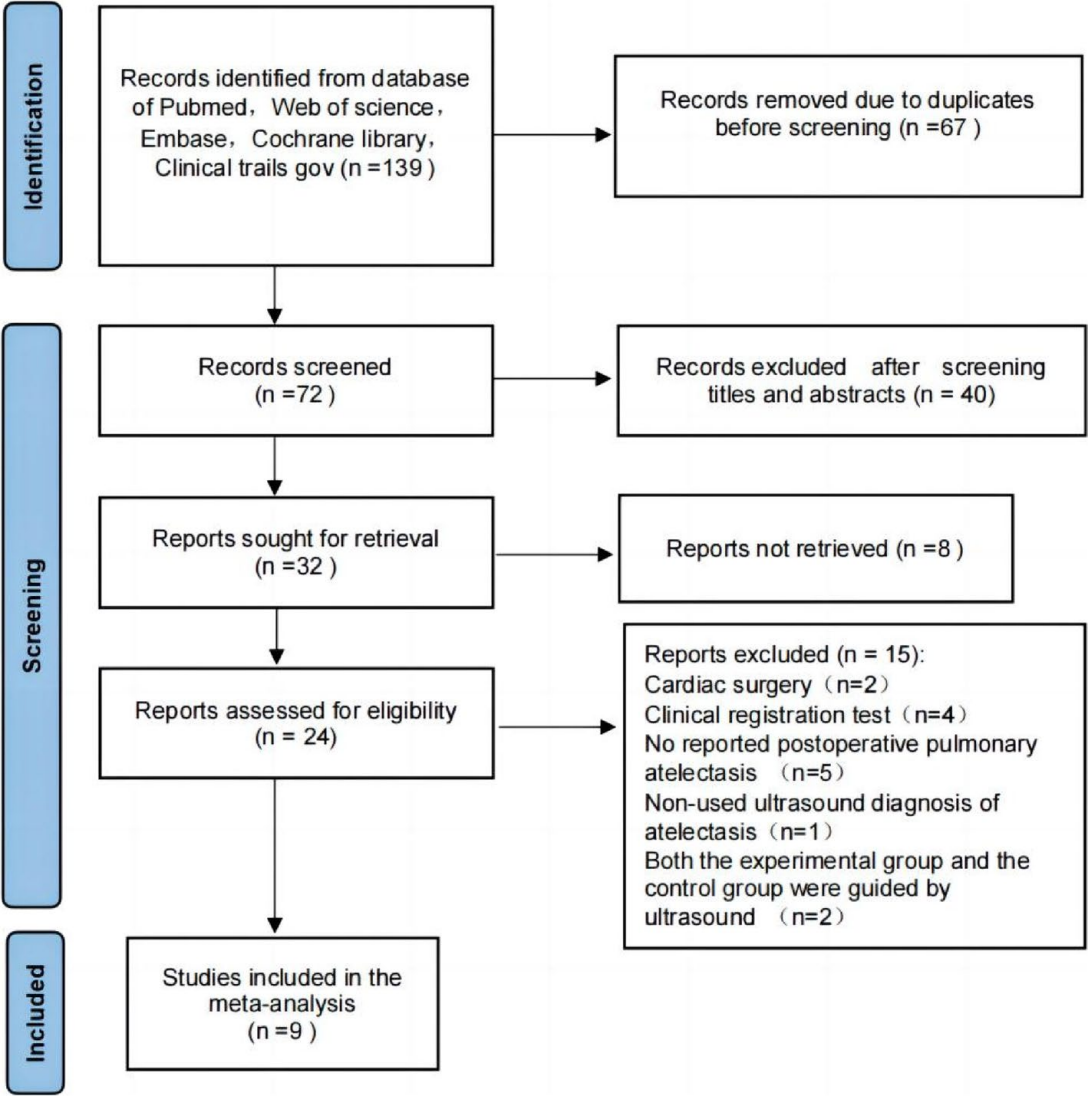
Flow chart of study screening

Figure 1 illustrates the systematic screening process conducted in this study. Initially, we searched 139 potentially relevant studies (PubMed, 21; Web of Science, 31; Embase, 27; Cochrane Library database, 46; Clinical Trials gov.14). After removing 67 duplicate studies, the remaining documents were subjected to comprehensive title and abstract screening. Subsequently, 48 studies that were deemed irrelevant, such as those that were non-randomized controlled trials, reviews, animal experiments, and those with inconsistent research purposes, were excluded. The articles were further assessed for eligibility, and 24 full texts were shortlisted. Ultimately, only 9 studies satisfied the inclusion criteria and were considered for meta-analysis. The reasons for excluding the remaining 15 studies were as follows: 4 were clinical registration trials, 2 were cardiac surgery, 5 did not report the results of postoperative atelectasis, 1 reported atelectasis was diagnosed by chest radiograph instead of lung ultrasound, and 3 performed ultrasound-guided or ultrasound-assessed LRM on both the experimental group and the control group. Finally, 9 randomized controlled trials were included in 443 patients (Acosta et al. 2018; Park et al. 2021; Lee et al. 2020; Song et al. 2017; Liu et al. 2022; Jang et al. 2020; Yang et al. 2021; Acosta et al. 2021; Acosta et al. 2020). The detailed information of the included study is shown in Table 2.

**Table 2:** Study characteristics

Study	Surgery	Age (years)	No.of patients (male/female)	ASA	FiO_2_(%)	TV (ml/kg)	PEEP (cmH_2_O)	LRM (Ultrasound-guided)	LRM (Control)
Lee 2020	Simple, superficial surgeries	≤ 6 years	86 (34/52)	*N*	0.4	8	5	The recruitment maneuver was performed under ultrasound guidance,a steady airway pressure of 15 cmH_2_O, with 5 cmH_2_O increments in PEEP until a peak pressure of 30 cmH_2_O was achieved	The recruitment maneuver was performed by maintaining a steady airway pressure of 15 cmH_2_O, with 5 cmH_2_O increments in PEEP until a peak pressure of 30 cmH_2_O was achieved
Song 2017	Elective minor surgery	≤ 1 years	40 (25/15)	I–II	0.4	8	5	The recruitment maneuver was performed after each lung ultrasound examination; a stepwise increase in airway pressure from 10 cmH_2_O by 5 cmH_2_O increments was applied manually	None
Park 2021	Laparoscopic gynaecological surgery	*N*	40 (0/40)	I–II	0.4	8	5	Manual inflation was applied until no collapsed areas were visible on the ultrasound	The recruitment maneuver was performed by manual inflation with a pressure of 30 cmH_2_O for 10 s
Liu 2022	Laparoscopic gynecologic surgery	18–65 years	41 (0/41)	I–II	0.4	6–8	6	The maximum airway pressure was set to start at 10 cmH_2_O and gradually increased by 5 cmH_2_O until the collapsed lung area was not visible on ultrasound	None
Jang 2020	Elective non-cardiac surgery in the prone position	< 3 years	40 (20/20)	*N*	0.4	6	7	Alveolar recruitment to restore FRC with 30–40 cmH_2_O of continuous positive airway pressure via a closed system for approximately 5–10 s	None
Yang 2021	Laparoscopic surgery for colorectal carcinoma	≥ 60 years	40 (30/10)	I–III	0.4	6–8	4	The recruitment maneuver was performed with a gradual rise in airway pressure from 10 cmH_2_O to 5 cmH_2_O increments and it was applied manually until no collapsed lung areas were visible on the sonogram	None
Acosta 2018	Non-emergency and non-thoracic surgery	6 months–7 years	42 (36/6)	I–II	0.5	6	5	The recruitment maneuver was performed after a lung ultrasound examination.PEEP was increased in steps of 5 cmH_2_O, from 5 to 15 cmH_2_O, every three breaths. The target recruitment pressure of 30 cmH_2_O was maintained for 10 breaths	None
Acosta 2021	Non-emergency and non-thoracic surgery	6 months–7 years	41 (26/15)	I	0.4	7	5	The recruitment maneuver was performed after a lung ultrasound examination.PEEP was increased in steps of 5 cmH_2_O, from 5 to 15 cmH_2_O, every three breaths. The target recruitment pressure of 30 cmH_2_O was maintained for 10 breaths	None
Acosta 2020	Non-emergency and non-thoracic surgery	6 months–5 years	40 (27/13)	I–II	0.5	6	5	Ventilation was turned to pressure control ventilation using a driving pressure of 12 cmH_2_O and PEEP of 10 cmH_2_O, but they were immediately and sequentially placed: (1) in the left lateral position (90 s), (2) in the right lateral position (other 90 s), (3) back to the supine position	None

*SD* standard deviation, *ASA* American Society of Anesthesiologists, *BMI* body mass index, *PEEP* positive end-expiratory pressure, *C* control, *LRM* lung recruitment maneuver, *TV* tidal volume, *FiO*_*2*_ fraction of inspired oxygen, *FRC* functional residual capacity, *CPAP* continuous positive airway pressure, *N* not reported

Table 2 outlines the fundamental characteristics of the included study. We included RCTs with ultrasound-guided LRM. These investigations need to use lung ultrasonography to evaluate atelectasis, count the number of cases in the experimental and control groups, and determine whether the difference is statistically significant. Our study defined the experimental group as those receiving ultrasound-guided LRM.

**Fig. 2:**
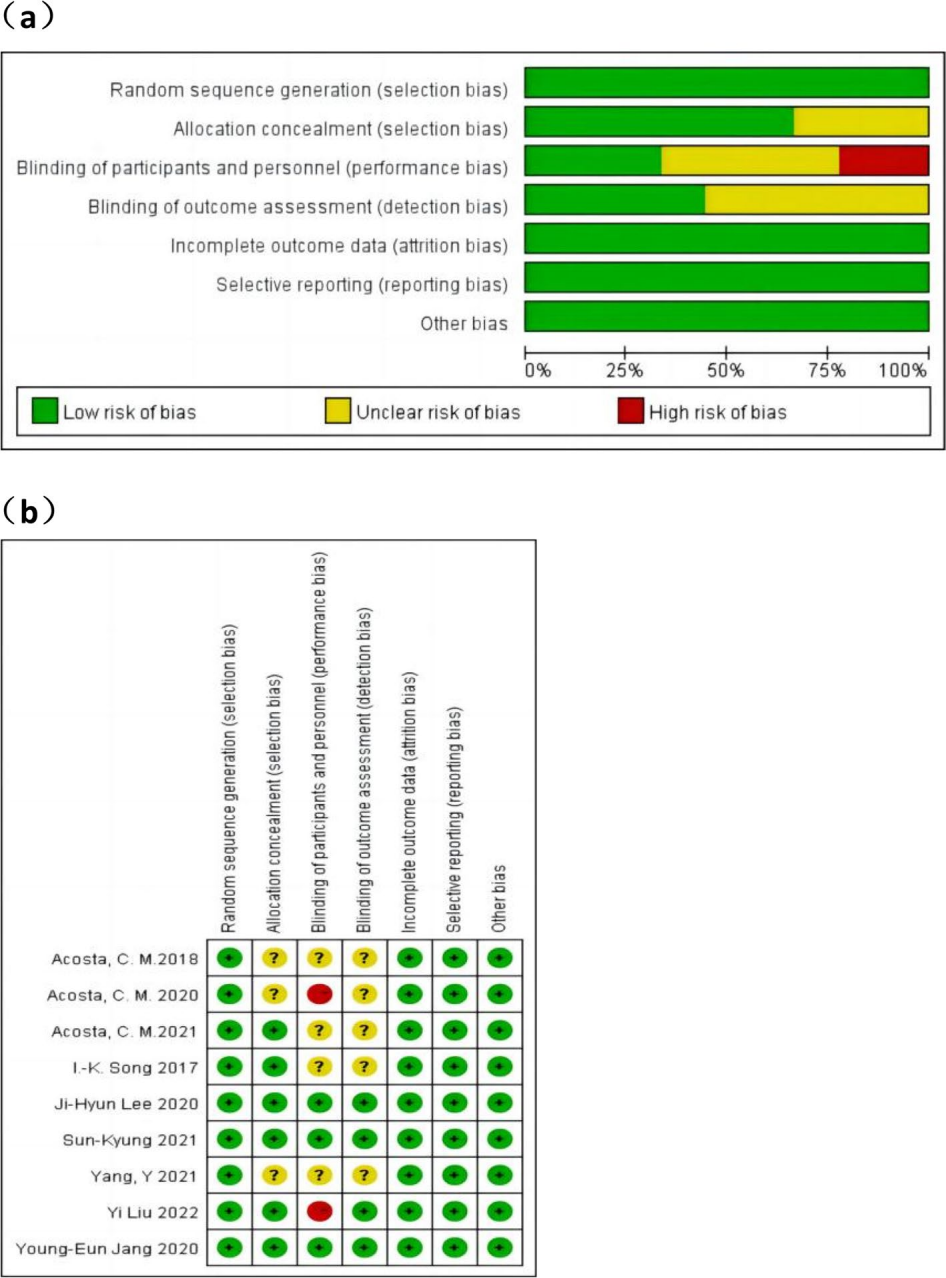
Evaluation of risk bias for included RCTs: **a** percentage plot of seven types of bias for the included studies; **b** summary of bias for each study

Figure 2 presents the results of the quality evaluation conducted using Review Manager 5.3. We assessed the integrity of outcome data and the risk of selective reporting of research results as low in all trials, indicating low risk in random sequence generation. However, Three trials (Acosta et al. 2018; Yang et al. 2021; Acosta et al. 2020) did not provide information about random assignment concealment. Additionally, two studies (Liu et al. 2022; Acosta et al. 2020) did not apply blinding measures to the researcher or the subject, while four studies (Acosta et al. 2018; Song et al. 2017; Yang et al. 2021; Acosta et al. 2021) omitted descriptions of whether blinding procedures were implemented for the researcher or the subject. Furthermore, five studies (Acosta et al. 2018; Song et al. 2017; Yang et al. 2021; Acosta et al. 2021; Acosta et al. 2020) failed to report whether the outcome evaluator was blinded. Nonetheless, nine studies (Acosta et al. 2018; Park et al. 2021; Lee et al. 2020; Song et al. 2017; Liu et al. 2022; Jang et al. 2020; Yang et al. 2021; Acosta et al. 2021; Acosta et al. 2020) pose a low risk of other biases.

### Grading evidence quality

The evaluation of the quality of evidence using GRADEpro is presented in Table 3. The assessment is based on several key parameters such as risks of bias, inconsistency, indirection, imprecision, and publication bias, and the evidence is subsequently categorized into four categories: high, medium, low, and extremely low. The risk of bias was evaluated by considering a total of 18 indicators, all of which were assessed as not serious. Due to *I*^2^, the inconsistency of LUS and LUS of each part were rated as serious > 50%, indicating unacceptable heterogeneity.

**Table 3:** Quality of evidence by GRADE

Certainty assessment							№ of patients		Effect		Certainty	Importance
№ of studies	Study design	Risk of bias	Inconsistency	Indirectness	Imprecision	Other considerations	Ultrasound-guided	Control	Relative (95% CI)	Absolute (95% CI)		
Incidence of atelectasis												
9	Randomized trials	Not serious	Not serious	Not serious	Not serious	None	52/222 (23.4%)	169/221 (76.5%)	RR 0.31 (0.25 to 0.40)	528 fewer per 1000 (from 574 to 459 fewer)	⨁⨁⨁⨁ High	Critical
Subgroup analysis of atelectasis by age												
9	Randomized trials	Not serious	Not serious	Not serious	Not serious	None	52/222 (23.4%)	169/221 (76.5%)	RR 0.31 (0.25 to 0.40)	528 fewer per 1000 (from 574 to 459 fewer)	⨁⨁⨁⨁ High	Critical
Subgroup analysis of atelectasis by age—age ≥ 18 (adult)												
3	Randomized trials	Not serious	Not serious	Not serious	Not serious	None	25/60 (41.7%)	52/61 (85.2%)	RR 0.49 (0.36 to 0.67)	435 fewer per 1000 (from 546 to 281 fewer)	⨁⨁⨁⨁ High	Critical
Subgroup analysis of atelectasis by age—age < 18 (children)												
6	Randomized trials	Not serious	Not serious	Not serious	Not serious	None	27/162 (16.7%)	117/160 (73.1%)	RR 0.23 (0.17 to 0.33)	563 fewer per 1000 (from 607 to 490 fewer)	⨁⨁⨁⨁ High	Critical
Subgroup analysis of atelectasis by LRM or Non-LRM used in the control group												
9	Randomized trials	Not serious	Not serious	Not serious	Not serious	None	52/222 (23.4%)	169/221 (76.5%)	RR 0.31 (0.25 to 0.40)	528 fewer per 1000 (from 574 to 459 fewer)	⨁⨁⨁⨁ High	Critical
Subgroup analysis of atelectasis by LRM or non-LRM used in the control group-compare to LRM in the control group												
3	Randomized trials	not serious	Not serious	Not serious	Not serious	None	14/100 (14.0%)	59/99 (59.6%)	RR 0.24 (0.14 to 0.39)	453 fewer per 1000 (from 513 to 364 fewer)	⨁⨁⨁⨁ High	Critical
Subgroup analysis of atelectasis by LRM or non-LRM used in the control group-compare to non-LRM in the control group												
6	Randomized trials	Not serious	Not serious	Not serious	Not serious	None	38/122 (31.1%)	110/122 (90.2%)	RR 0.35 (0.27 to 0.46)	586 fewer per 1,000 (from 658 to 487 fewer)	⨁⨁⨁⨁ High	Critical
The application of PEEP after LRM												
9	Randomized trials	Not serious	Not serious	Not serious	Not serious	None	52/222 (23.4%)	169/221 (76.5%)	RR 0.31 (0.25 to 0.40)	528 fewer per 1,000 (from 574 to 459 fewer)	⨁⨁⨁⨁ High	Critical
The application of PEEP after LRM-PEEP(ultrasound-guided) = PEEP (control)												
7	Randomized trials	Not serious	Not serious	Not serious	Not serious	None	41/180 (22.8%)	131/180 (72.8%)	RR 0.32 (0.24 to 0.42)	495 fewer per 1000 (from 553 to 422 fewer)	⨁⨁⨁⨁ High	Critical
The application of PEEP after LRM-PEEP (ultrasound-guided) > PEEP (control)												
2	Randomized trials	Not serious	Not serious	Not serious	Not serious	None	11/42 (26.2%)	38/41 (92.7%)	RR 0.29 (0.18 to 0.48)	658 fewer per 1000 (from 760 to 482 fewer)	⨁⨁⨁⨁ High	Critical
LUS after LRM												
6	Randomized trials	Not serious	Not serious	Not serious	Not serious	None	122	122	-	MD 6.24 lower (6.9 lower to 5.59 lower)	⨁⨁⨁⨁ High	Critical
LUS of each part of the lung after LRM												
2	Randomized trials	Not serious	Not serious	Not serious	Not serious	None	120	123	–	MD **2.31** lower (2.69 lower to 1.94 lower)	⨁⨁⨁⨁ High	Critical
LUS of each part of the lung after LRM–anterior												
2	Randomised trials	Not serious	Not serious	Not serious	Not serious	None	40	41	–	MD 2 lower (2.49 lower to 1.51 lower)	⨁⨁⨁⨁ High	Critical
LUS of each part of the lung after LRM–lateral												
2	Randomised trials	Not serious	Not serious	Not serious	Not serious	None	40	41	–	MD **2.5 lower** (3.2 lower to 1.8 lower)	⨁⨁⨁⨁ High	Critical
LUS of each part of the lung after LRM-posterior												
2	Randomized trials	Not serious	Not serious	Not serious	Not serious	None	40	41	–	MD 3.24 lower (4.23 lower to 2.24 lower)	⨁⨁⨁⨁ High	Critical
Certainty assessment							№ of patients		Effect		Certainty	Importance

*CI* confidence interval, *MD* mean difference, *RR* risk ratio

In terms of indirectness and imprecision, since all studies directly compared ultrasound-guided LRM with a certain sample size and a control group, the indicators were classified as non-serious. Given the aforementioned assessment, we can confidently state high confidence in all the results.

### Primary outcomes

#### Incidence of postoperative atelectasis

**Fig. 3:**
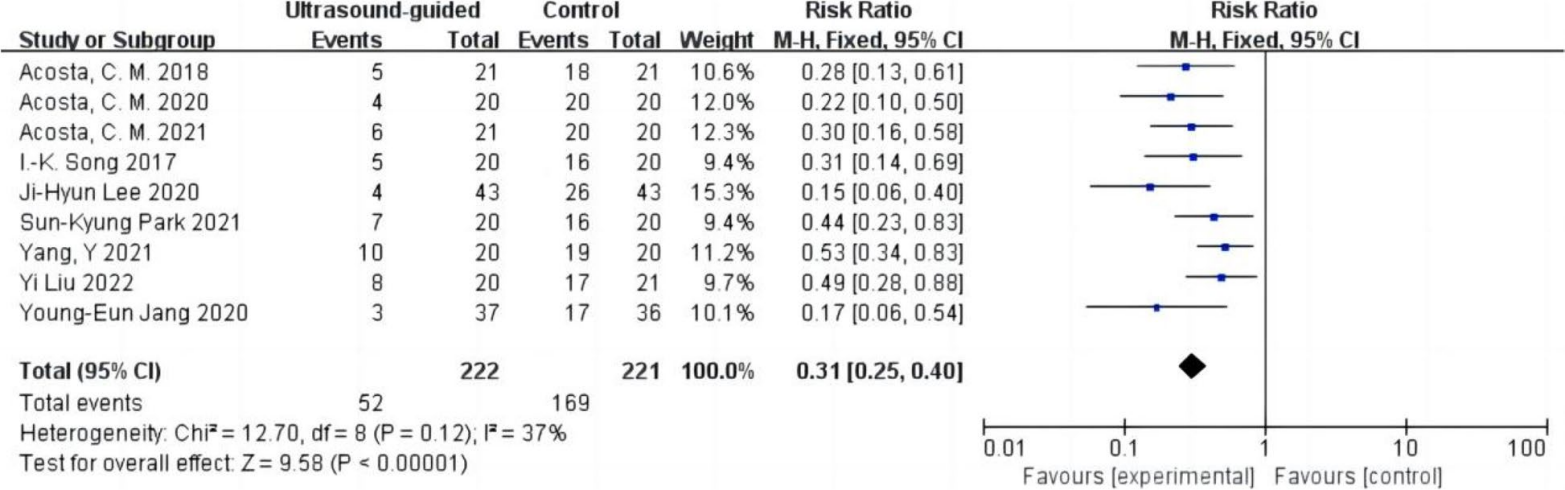
Forest plot for the incidence of postoperative atelectasis between the ultrasound-guided and control groups. CI = confidence interval, RR = risk ratio, M-H = methods of merging dichotomous variables

The incidence of postoperative atelectasis was reported in nine studies with 443 patients (Acosta et al. 2018; Park et al. 2021; Lee et al. 2020; Song et al. 2017; Liu et al. 2022; Jang et al. 2020; Yang et al. 2021; Acosta et al. 2021; Acosta et al. 2020). Among them, there were 222 cases in the ultrasound-guided LRM group and 221 cases in the control group (Fig. 3). The incidence of postoperative atelectasis in the ultrasound-guided LRM group was lower than in the control group. Low heterogeneity was observed in the results (RR 0.31,95% CI 0.25 to 0.40,*p* < 0.05,heterogeneity *P* > 0.10,*I*^2^ = 37%).

#### Subgroup analysis of postoperative atelectasis by LRM or non-LRM used in the control group

**Fig. 4:**
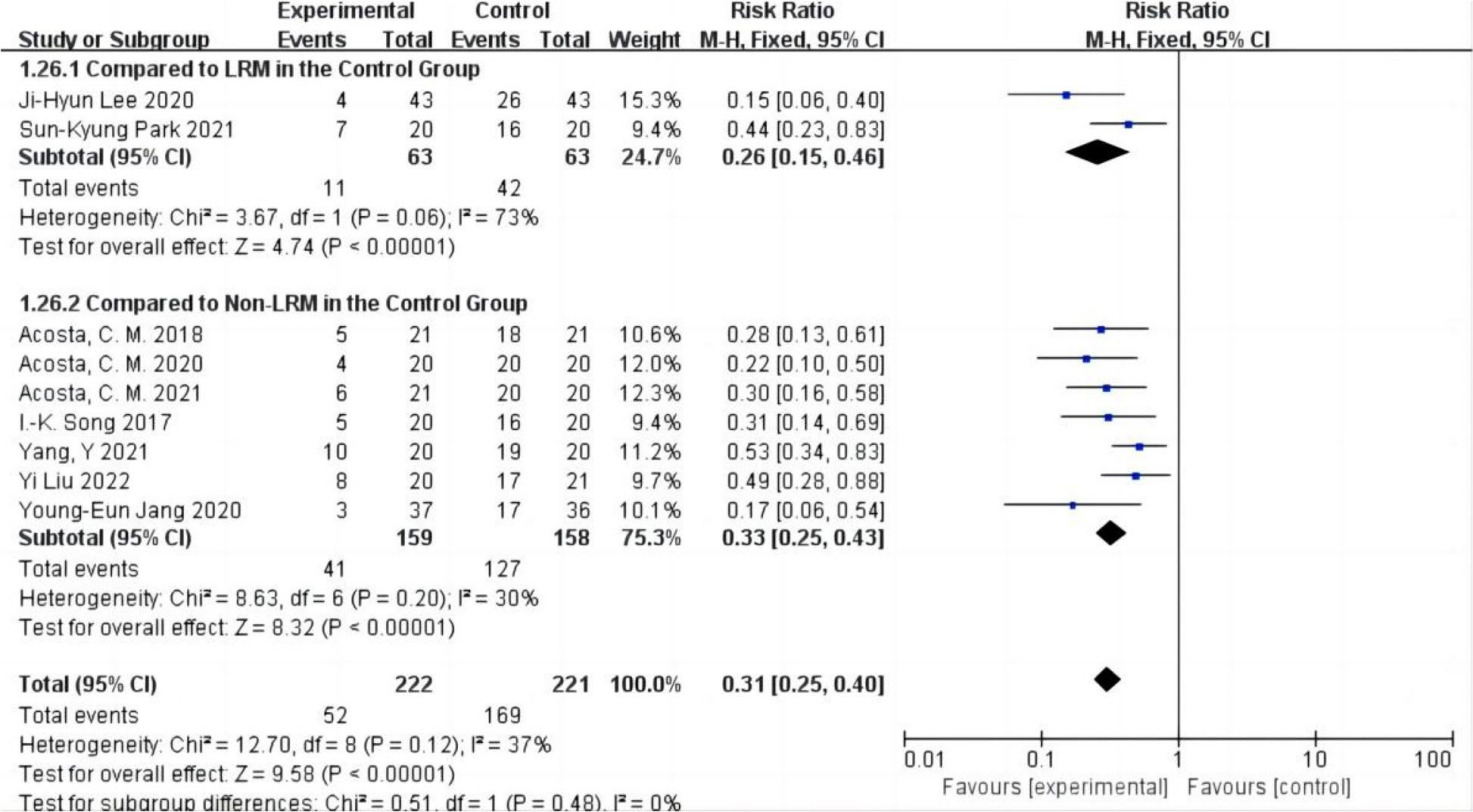
Forest plot for subgroup analysis of the incidence of postoperative atelectasis between the ultrasound-guided and control groups. Grouped by LRM or non-LRM used in the control group: compared to LRM in the control group, compared to non-LRM in the control group. CI = confidence interval, RR = risk ratio, M-H = methods of merging dichotomous variables

Whether the control group used LRM or not was reported in the nine studies with 443 patients (Acosta et al. 2018; Park et al. 2021; Lee et al. 2020; Song et al. 2017; Liu et al. 2022; Jang et al. 2020; Yang et al. 2021; Acosta et al. 2021; Acosta et al. 2020), of which 126 patients in the control group used LRM in two studies (Park et al. 2021; Lee et al. 2020), and 317 patients in five studies did not use LRM (Acosta et al. 2018; Song et al. 2017; Liu et al. 2022; Jang et al. 2020; Yang et al. 2021; Acosta et al. 2021; Acosta et al. 2020). The findings demonstrated that whether the control group did not employ LRM or did so with non-ultrasound-guided LRM, the incidence of postoperative atelectasis was reduced in patients with ultrasound-guided LRM (compared to non-LRM in the control group: RR = 0.33,95% CI 0.25–0.43,*P* < 0.05,heterogeneity *p* > 0.10,*I*^2^ = 30%,compared to LRM in the control group: RR = 0.26,95% CI 0.15–0.46,*P* < 0.05,heterogeneity *P* < 0.10,*I*^2^ = 73%) (Fig. 4).

#### Subgroup analysis of the effect of adults and children on postoperative atelectasis

**Fig. 5:**
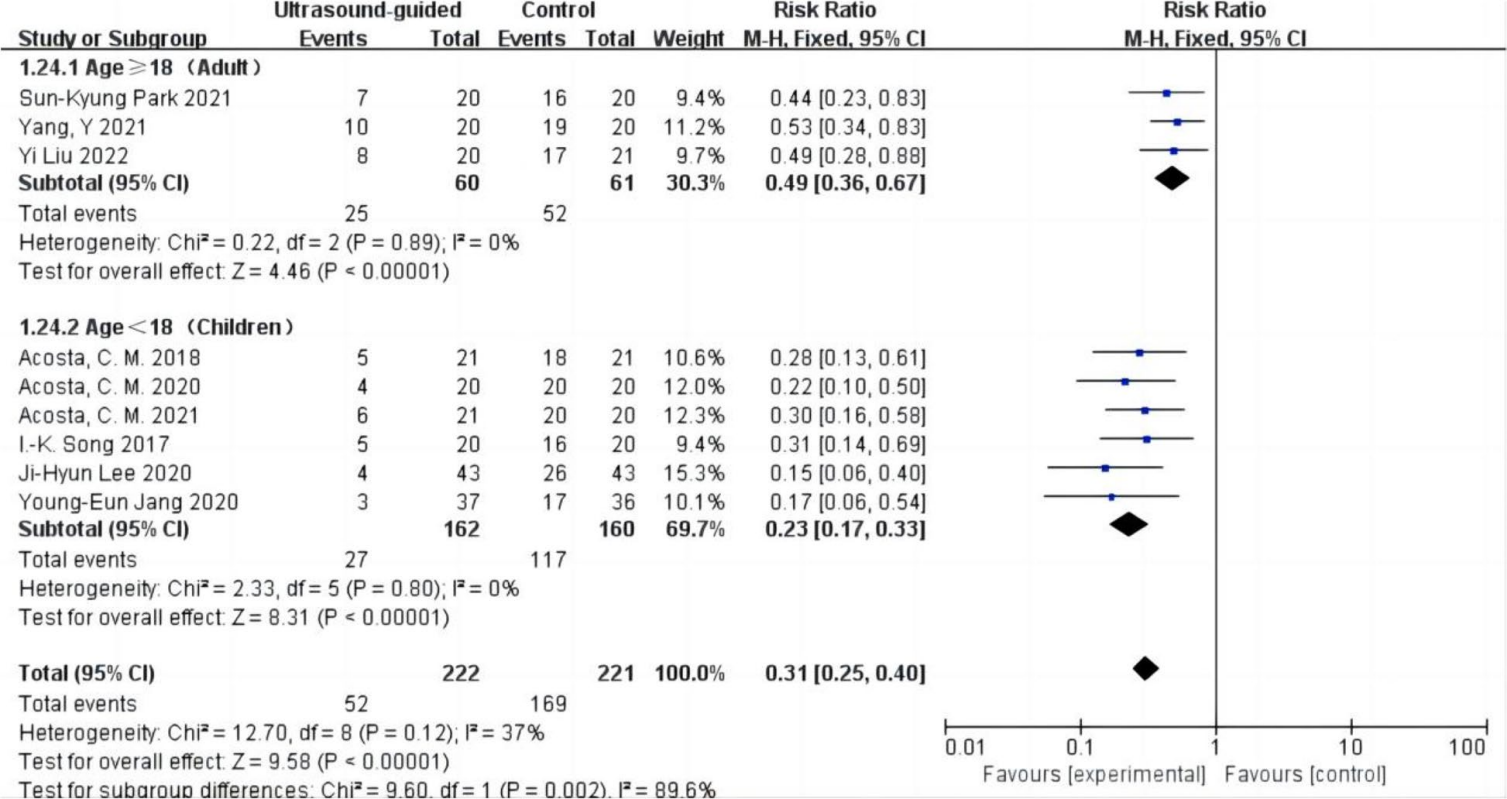
Forest plot for subgroup analysis of the incidence of postoperative atelectasis between the ultrasound-guided and control groups. Grouped by age: age ≥ 18 years (adult), age < 18 years (children). CI = confidence interval, RR = risk ratio, M-H = methods of merging dichotomous variables

The incidence of postoperative atelectasis in adults and children was reported in the nine studies with 443 patients (Acosta et al. 2018; Park et al. 2021; Lee et al. 2020; Song et al. 2017; Liu et al. 2022; Jang et al. 2020; Yang et al. 2021; Acosta et al. 2021; Acosta et al. 2020). The results showed that ultrasound-guided LRM reduced the incidence of postoperative atelectasis in adults (RR = 0.49,95% CI 0.36 to 0.67,*p* < 0.05,heterogeneity *p* > 0.10,*I*^2^ = 0%) (Fig. 5). Using ultrasound-guided LRM also reduces the incidence of postoperative atelectasis in children (RR = 0.23,95% CI 0.17 to 0.33,*p* < 0.05,heterogeneity *p* > 0.10,*I*^2^ = 0%). It may be more effective in children than adults (heterogeneity *p* < 0.05,*I*^2^ = 89.6%; *P* for subgroup differences < 0.01).

#### Subgroup analysis was performed in the present study to investigate the effect of PEEP after LRM on postoperative atelectasis

**Fig. 6:**
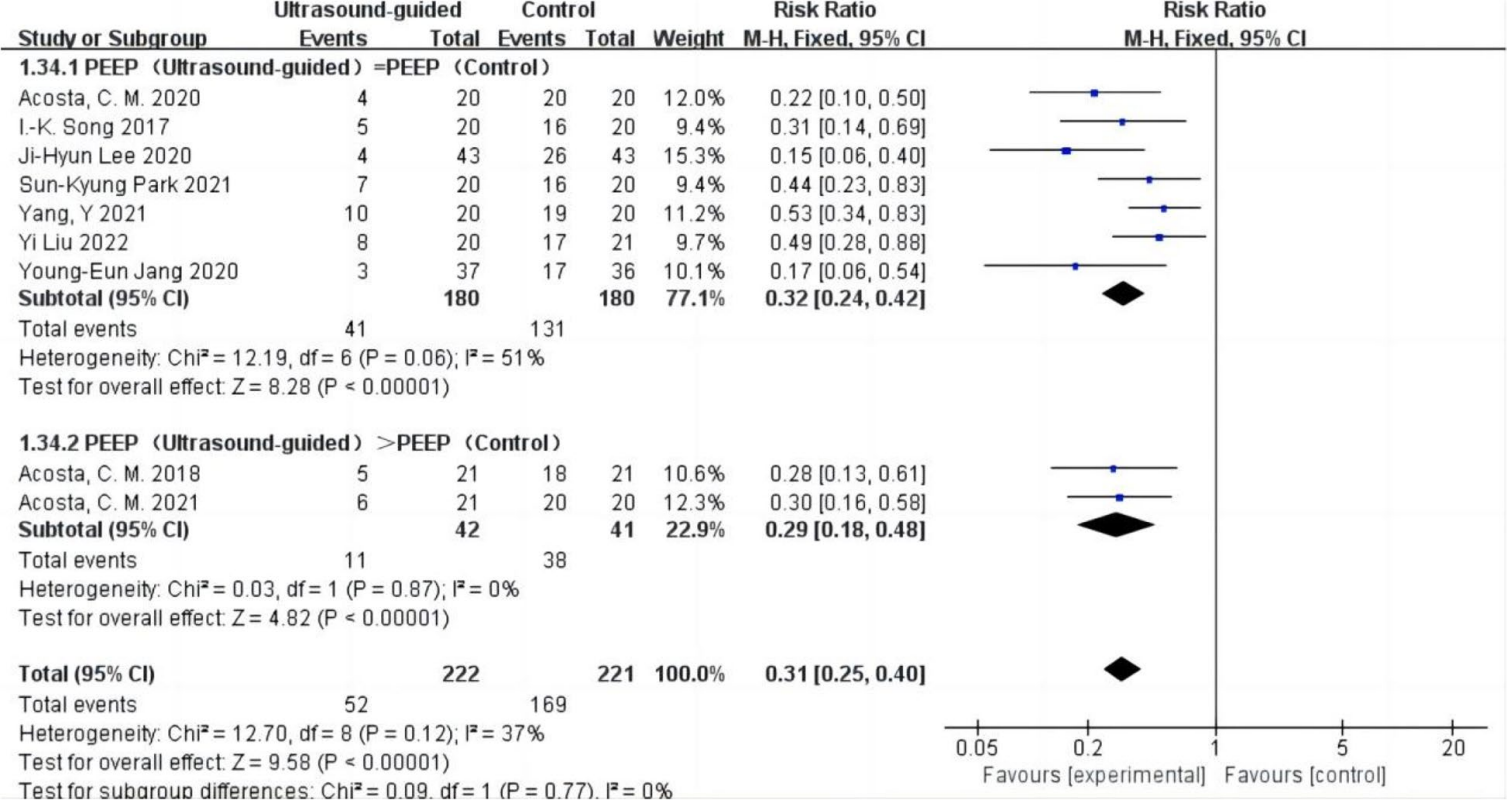
Forest plot for subgroup analysis of the incidence of postoperative atelectasis between the ultrasound-guided and control groups. Grouped by PEEP after LRM: PEEP (ultrasound-guided) = PEEP (control), PEEP (ultrasound-guided) > PEEP(Control). PEEP = positive end-expiratory pressure, CI = confidence interval, RR = risk ratio, M-H = methods of merging dichotomous variables

In 9 studies, 443 patients (Acosta et al. 2018; Park et al. 2021; Lee et al. 2020; Song et al. 2017; Liu et al. 2022; Jang et al. 2020; Yang et al. 2021; Acosta et al. 2021; Acosta et al. 2020) reported the incidence of postoperative atelectasis with PEEP after LRM. Among 7 studies, 360 patients maintained the same PEEP before and after LRM (Park et al. 2021; Lee et al. 2020; Song et al. 2017; Liu et al. 2022; Jang et al. 2020; Yang et al. 2021; Acosta et al. 2020), while the remaining 2 studies adjusted for PEEP after LRM according to ultrasound guidance, which resulted in a higher PEEP compared to the control group (Acosta et al. 2018; Acosta et al. 2021). The pooled incidence of postoperative atelectasis was lower in patients who underwent ultrasound-guided LRM than those who received non-ultrasound-guided LRM (RR = 0.31,95% CI 0.25 to 0.40,*p* < 0.05,heterogeneity *p* > 0.10,*I*^2^ = 37%) (Fig. 6). There is a slight advantage in combining high PEEP after LRM compared to combining low PEEP (heterogeneity *p* > 0.10,*I*^2^ = 0%,*P* for subgroup differences < 0.01). However, due to the lack of research on the use of higher PEEP after non-ultrasound-guided LRM compared to ultrasound-guided LRM, no definitive conclusion could be drawn based on the available data.

### Secondary outcomes

#### LUS

**Fig. 7:**
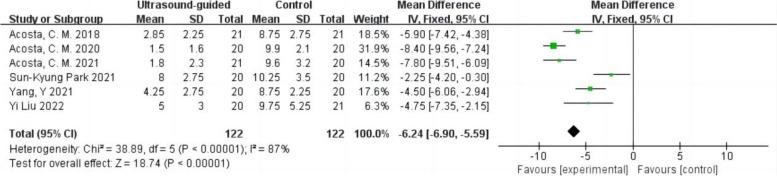
Forest plot for the LUS between the ultrasound-guided and control groups. CI = confidence interval, IV = inverse variance

The LUS after surgery was reported in six studies with 244 patients (Acosta et al. 2018; Park et al. 2021; Liu et al. 2022; Yang et al. 2021; Acosta et al. 2021; Acosta et al. 2020), and the other three reported consolidation and B-line scores (Lee et al. 2020; Song et al. 2017; Jang et al. 2020). The LUS after surgery was statistically different between the ultrasound-guided LRM and the control group (Fig. 7). The LUS after surgery in the ultrasound-guided LRM group was lower. High heterogeneity was observed in the results (WMD –6.24,95% CI –6.90 to − 5.59,*p* < 0.05,heterogeneity *p* < 0.10,*I*^2^ = 87%).

#### LUS of each part

**Fig. 8:**
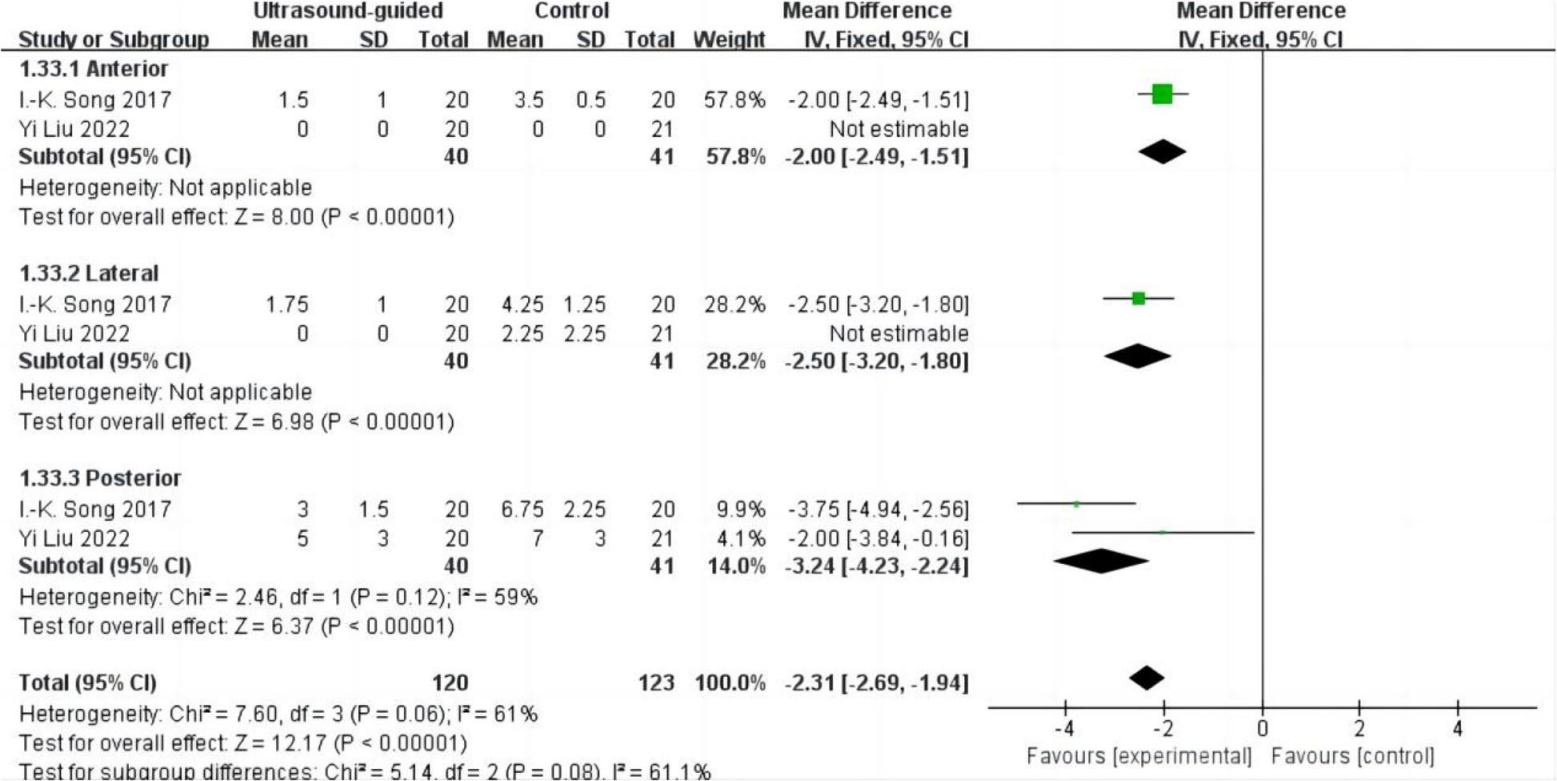
Forest plot for the LUS of each part between the ultrasound-guided and control groups. CI = confidence interval, IV = inverse variance

Two studies, including 81 patients, reported LUS of each part after surgery (Song et al. 2017; Liu et al. 2022). After the surgery, the LUS of each part is as follows: LUS in the anterior lung region (WMD − 2.00,95% CI − 2.49 to − 1.51,*p* < 0.05),LUS in lateral lung region (WMD − 2.50; 95% CI − 3.20 to − 1.80; *p* < 0.05); LUS in the posterior lung region (WMD − 3.24; 95% CI − 4.23 to − 2.24; *p* < 0.05; heterogeneity *p* > 0.10; *I*^2^ = 59%) (Fig. 8). The results showed that compared with the control group, the ultrasound-guided group could reduce the LUS of the anterior, lateral, and posterior parts, and the results were statistically significant.

### Publication bias

**Fig. 9:**
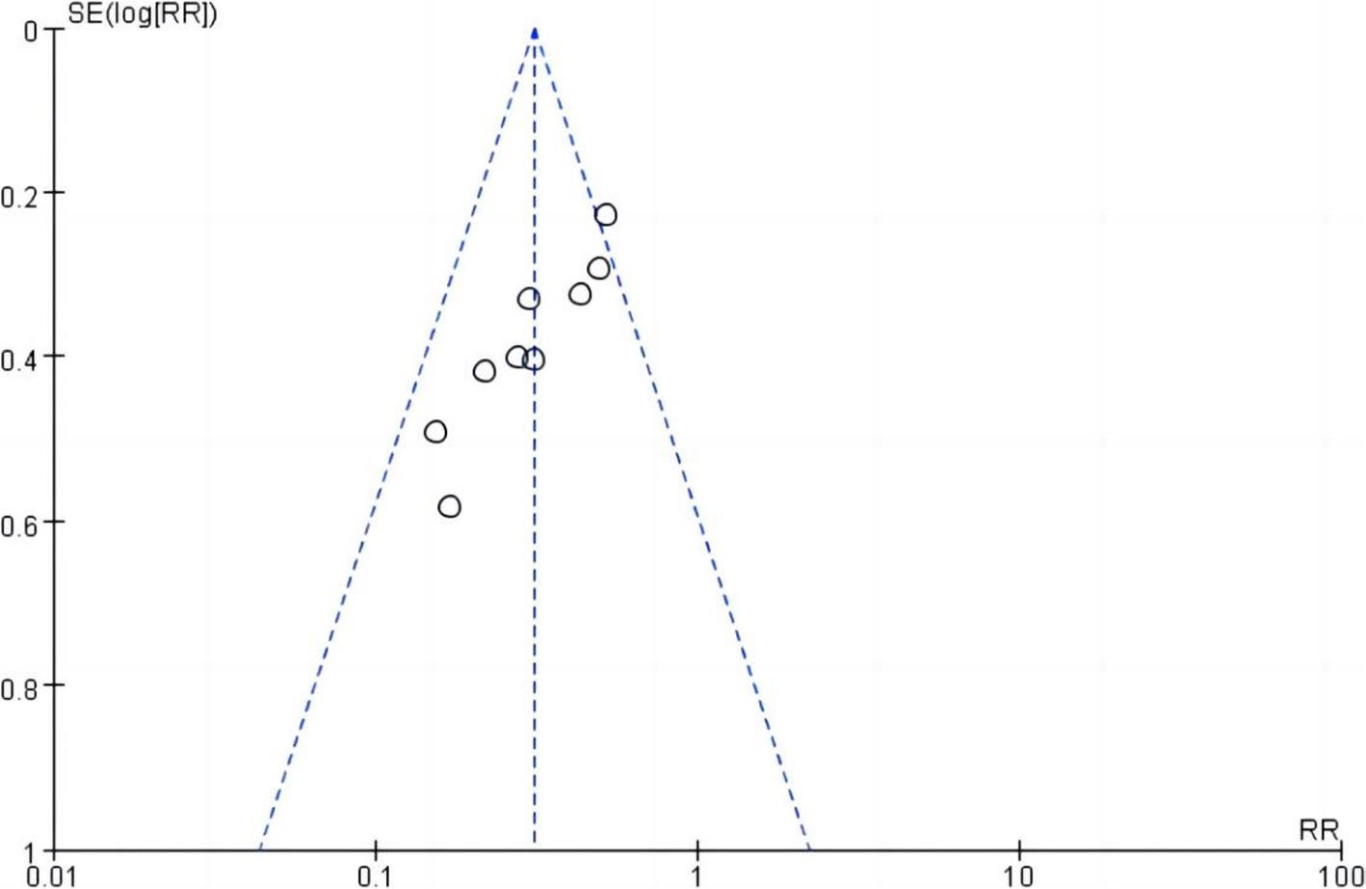
Funnel plot for the incidence of postoperative atelectasis

The funnel plot of primary outcomes is presented in Fig. 9 (Incidence of postoperative atelectasis). The funnel plot was used to evaluate the publication bias of the included study. Visual inspection of the funnel plot found no evidence of publication bias in the primary outcome. Combined with the funnel plot results, the results of the Egger test showed that the Egger test *t* = 0.16, *P* >| *t* |= 0.874 (*P* > 0.05) suggested there was little possibility of publication bias in the nine articles included.

## Discussion

This meta-analysis aimed to compare the efficacy of the ultrasound-guided lung recruitment maneuver (LRM) strategy with the non-ultrasound-guided ventilation strategy in reducing postoperative atelectasis in patients undergoing non-cardiac surgery. Despite the recognized effectiveness of LRM in reducing postoperative atelectasis, there is a paucity of systematic evaluations or meta-analyses that report the impact of ultrasound-guided LRM on patients. Therefore, it is imperative to comprehensively analyze the existing randomized controlled trials to establish the benefits of this technique. The results of our analysis demonstrate that ultrasound-guided LRM is superior to the non-ultrasound-guided ventilation strategy in reducing postoperative atelectasis and improving lung aeration. The heterogeneity of LUS is high, while the incidence of atelectasis is low. The heterogeneity of LUS may come from several sources. First, the enrolled patients have a wide range of ages and different operations. Secondly, the intraoperative ventilation strategy is highly variable. Tidal volume, LRM, and PEEP can affect oxygenation and respiratory mechanics, resulting in differences in LUS changes after LRM.

In terms of the effectiveness of ultrasound-guided LRM, according to the study of Monassese et al. (Monastesse et al. 2017), the LUS in the lung ultrasound image is significantly related to the degree of ventilation function damage and the number of atelectasis areas. According to the results of this study, the LUS of the ultrasound-guided LRM group decreased by 6.24 points on average compared with the non-ultrasound control group after the surgery, and the number of atelectasis in the ultrasound-guided LRM group (23.4%) after the surgery was significantly lower than that in the non-ultrasound control group (76.5%). The above results also help to confirm the good consistency between LUS and the diagnosis of atelectasis. Among the LUS of atelectasis sites, the LUS of the posterior lung region is the highest, and the effect of LRM is the most significant. The findings support the idea that atelectasis occurs in gravity-dependent regions (Tusman et al. 2003), and ultrasound-guided LRM lowers the LUS of the posterior lung area by an average of 3.24 points, which is significantly better than that of the non-ultrasound control group. This finding supports the idea that lung ultrasound has some advantages in visualizing and purposefully guiding lung recruitment strategy. The technique of ultrasound-guided LRM can dramatically lower the frequency of postoperative atelectasis during surgery and lessen the severity of atelectasis, according to the aforementioned findings.

Despite the significant reduction in postoperative atelectasis incidence following ultrasound-guided LRM in both children and adult subgroups, the overall risk of atelectasis in children was found to be significantly lower at 23% as compared to 49% in adults. The following two factors should be considered: Firstly, Yang Y et al. (Yang et al. 2021) in the adult subgroup are laparoscopic surgery for the elderly, and Yi Liu et al. (Liu et al. 2022) are also included in laparoscopic surgery for some elderly patients,Secondly, in the adult subgroup, the operation is more complicated, resulting in longer mechanical ventilation time. “*Lung-protective ventilation for the surgical patient: international expert panel-based consensus recommendations*” (Young et al. 2019) published in 2019 indicates that age > 50 years old and mechanical ventilation time > 2 h are risk factors for atelectasis. Hence, the adult subgroup in the study has a higher risk of postoperative atelectasis. It also shows the necessity of LRM in such operations. It is also anticipated that future pertinent studies will provide more conclusive results to confirm the viability of ultrasound-guided surgery in the elderly population because most current studies concentrate on ultrasound-guided LRM in children, while very few studies are conducted on the elderly population at high risk of postoperative atelectasis.

Further discussion is merited regarding the use of a control group with LRM. Some studies employing low tidal volume and PEEP did not involve lung re-expansion during surgery in their control group (Acosta et al. 2018; Song et al. 2017; Liu et al. 2022; Jang et al. 2020; Yang et al. 2021; Acosta et al. 2021; Acosta et al. 2020), while other studies used non-ultrasound-guided LRM during surgery for their control group (Park et al. 2021; Lee et al. 2020). Despite differences in postoperative atelectasis incidence, the data demonstrated that the incidence of the condition was decreased in the ultrasound-guided group. Surprisingly, compared with the subgroup of the control group who underwent LRM, the patients who did not undergo LRM had a lower risk of postoperative atelectasis. Ji-Hyun Lee et al. (Lee et al. 2020) found that even with the use of LRM, alveolar atelectasis regression could not be guaranteed at an airway pressure of 30 cm H_2_O. Most patients in the ultrasound-guided group required pressures exceeding 30 cm H_2_O. Most patients in the ultrasound-guided group needed more than 30 cm H_2_O pressure to make the alveolar re-expansion. Therefore, the two studies, including LRM in the control group, limited the airway pressure below 30 cm H_2_O, which also limited the advantages of LRM, and verified that ultrasound-guided high-quality LRM is an important means to ensure the effectiveness of LRM. In the past, there was no high-quality evidence to recommend routine LRM after tracheal intubation for patients undergoing general anesthesia. Anesthesiologists need to evaluate the risk–benefit ratio of patients in order to develop treatment plans. Blind lung recruitment may benefit patients less and have adverse effects. What helps to eliminate such concerns is the significance of ultrasound-guided lung recruitment.

Our investigation also examined the impact of positive end-expiratory pressure (PEEP) on postoperative atelectasis following lung resection surgery. The included studies all applied PEEP (≥ 4 cmH_2_O) throughout the surgery, and the results showed that using PEEP after ultrasound-guided LRM had a lower risk of postoperative atelectasis compared to using PEEP after non-ultrasound-guided LRM. Two studies appropriately increased PEEP after LRM (with a maximum PEEP of 8 cmH_2_O), with the aim of better maintaining lung expansion and avoiding further collapse. However, compared to maintaining the original PEEP after LRM, the use of higher levels of PEEP was associated with a lower risk of postoperative atelectasis. This is similar to the results of a previous meta-analysis (Campos et al. 2022), which included 3837 surgical patients who used different levels of PEEP combined with LRM during surgery. After exploring the impact of lung complications within 7 days after surgery, it was found that the high PEEP group had a lower incidence of postoperative atelectasis compared to the low PEEP group. However, it is worth mentioning that once confounding factors were adjusted for in this study, there was no significant difference in the incidence of postoperative atelectasis between the two PEEP levels. Although the meta-analysis of postoperative atelectasis was diagnosed through chest X-ray rather than lung ultrasound, it can be seen that the level of PEEP used after LRM is not clear which to prevent postoperative atelectasis in patients.

Studies have shown that in adults, lung ultrasound exhibits good sensitivity (87%), specificity (92%), and accuracy (91%) in verifying the occurrence of atelectasis compared to computed tomography (CT) scans (Yu et al. 2016), but lung ultrasound also has its limitations. Obese patients are frequently difficult to examine using lung ultrasound because of the thickness of subcutaneous tissue around the rib cage. The presence of subcutaneous emphysema or large thoracic dressings precludes the propagation of ultrasound beams to the lung periphery and makes lung ultrasound examination difficult (Bouhemad et al. 2011 Feb 1). During surgery, the fixation of body position is also one of the reasons for the limited use of lung ultrasound, such as difficulty in placing the ultrasound probe at the back of the patient in a supine position. In addition, studies have found that although ultrasound-guided LRM can improve ventilation in laparoscopic gynecological surgery, there is no statistically significant difference in respiratory mechanics and oxygenation compared to non-ultrasound-guided LRM (Park et al. 2021). Finally, although ultrasound-guided LRM can re-expand collapsed lungs, the inflated lung area may also have over-inflation during LRM.

This meta-analysis exhibits certain limitations that must be taken into consideration. ① The clinical heterogeneity among the included studies, such as variations in surgical type, anesthesia induction, mechanical ventilation time, and lung recruitment maneuver (LRM), poses a challenge in determining the optimum personalized lung protective ventilation strategy for specific populations and surgeries. Further data analysis based on factors such as PEEP level, the specific operation or patient population, and the duration of mechanical ventilation is required to establish definitive guidelines. However, there is still a dearth of high-quality studies available to aid in selecting the most personalized lung protective ventilation approach for various individuals and operations. Lung ultrasound has not yet found widespread use in the field of perioperative use. ② Low-risk patients recover from atelectasis quickly after short surgery, and it is uncertain whether the patients receiving short-term mechanical ventilation benefit from lung protective ventilation, including the use of low tidal volume, high level of PEEP, and/or various LRM during surgery. ③ The anesthesiologists, instead of trained ultrasound professionals, evaluated some studies to assess lung ultrasound, which could lead to errors in the evaluation results, considering the impact of anesthesiologists' limited technical expertise and experience. ④ The results of this meta-analysis are only limited to patients without lung disease, patients with high-risk and complicated lung disease, or emergency surgery, and further research is needed. ⑤ The RCTs included in this study are single-center and small-sample trials, which may have bias risk. In the future, multi-center and large-sample trials are needed to improve the analysis results.

## Conclusion

Ultrasound-guided lung recruitment maneuvers have been shown to be a promising approach for improving perioperative lung ventilation by increasing aeration while mitigating the development of atelectasis. In comparison to non-ultrasound-guided methods, this technique has exhibited superior effects.

### Supplementary Information

**Supplementary Material 1.**

## Data Availability

All data generated or analyzed during this study are included in this published article [and its supplementary information files].

## Abbreviations

LRM: Lung recruitment maneuvers
RCTs: Randomized controlled trials
LUS: Lung ultrasound score
PPCs: Postoperative pulmonary complications
MeSH: Medical Subject Headings
RR: Relative risk
PRISMA: Preferred Reporting Items for the Systematic Reviews and Meta-Analyses
MD: Mean differences
WMD: Weighted mean difference
SD: Standard deviation
ASA: American Society of Anesthesiologists
BMI: Body mass index
PEEP: Positive end-expiratory pressure
C: Control
TV: Tidal volume
FiO_2_: Fraction of inspired oxygen
FRC: Functional residual capacity
N: Not reported
CI: Confidence interval
